# Telomere-length dependent T-cell clonal expansion: A model linking ageing to COVID-19 T-cell lymphopenia and mortality

**DOI:** 10.1016/j.ebiom.2022.103978

**Published:** 2022-04-01

**Authors:** James J. Anderson, Ezra Susser, Konstantin G. Arbeev, Anatoliy I. Yashin, Daniel Levy, Simon Verhulst, Abraham Aviv

**Affiliations:** a School of Aquatic and Fishery Sciences, University of Washington, Seattle, WA 98195, USA; b Department of Epidemiology, Mailman School of Public Health, Columbia University, New York, NY 10032, USA; c New York State Psychiatric Institute, New York, NY 10032, USA; d Biodemography of Aging Research Unit, Social Science Research Institute, Duke University, Durham, NC 27705, USA; e Population Sciences Branch, National Heart, Lung, and Blood Institute, National Institutes of Health, Bethesda, MD 27705, USA; f The Framingham Heart Study, Framingham, MA 01702, USA; g Groningen Institute for Evolutionary Life Sciences, University of Groningen, Groningen, the Netherland; h The Center of Human Development and Aging, New Jersey Medical School, Rutgers University, Newark, NJ 07103, USA

**Keywords:** Telomeres, T-cells, COVID-19, SARS-CoV-2, Ageing, Vaccines

## Abstract

**Background:**

Severe COVID-19 T-cell lymphopenia is more common among older adults and entails poor prognosis. Offsetting the decline in T-cell count during COVID-19 demands fast and massive T-cell clonal expansion, which is telomere length (TL)-dependent.

**Methods:**

We developed a model of TL-dependent T-cell clonal expansion capacity with age and virtually examined the relation of T-cell clonal expansion with COVID-19 mortality in the general population.

**Findings:**

The model shows that an individual with average hematopoietic cell TL (HCTL) at age twenty years maintains maximal T-cell clonal expansion capacity until the 6th decade of life when this capacity rapidly declines by more than 90% over the next ten years. The collapse in the T-cell clonal expansion capacity coincides with the steep increase in COVID-19 mortality with age.

**Interpretation:**

Short HCTL might increase vulnerability of many older adults, and some younger individuals with inherently short HCTL, to COVID-19 T-cell lymphopenia and severe disease.

**Funding:**

A full list of funding bodies that contributed to this study can be found in the Acknowledgements section.

**Research in context**

**Evidence before this study**

We used the terms “telomere”, “COVID-19”, “SARS-CoV-2” for a search in PubMed and retrieved 37 papers. None of the papers examined nor considered the role of hematopoietic cell telomere length (HCTL) dynamics and T-cell clonal expansion in the pathogenesis of COVID-19.

**Added value of this study**

We integrate age-dependent HCTL shortening with T-cell clonal expansion into a model that links HCTL with T-cell lymphopenia and severe COVID-19 in older adults and individuals with inherently short telomeres.

**Implications of all the available evidence**

Our model suggests that short HCTL contributes to the development of COVID-19 T-cell lymphopenia— a potential mechanism linking telomere biology with COVID-19.

Alt-text: Unlabelled box

## Introduction

COVID-19 is confronting health-care workers with mortality from ageing-related diseases.^1^^,^^2^ Short telomere length (TL) in hematopoietic cells contributes to mortality from these diseases^3^ and it might play a role in severe COVID-19.^4^, ^5^, ^6^, ^7^ A study of adults whose hematopoietic cell TL (HCTL) had been measured several years before the COVID-19 pandemic observed that among individuals who went on to contract COVID-19, those with short HCTL were more likely to have severe disease.^7^ This finding is consistent with a causal role of HCTL in some ageing-related diseases.^8^^,^^9^ Although the mechanisms that link short HCTL with severe COVID-19 are partially understood, COVID-19 lymphopenia is a potential explanation.^10^, ^11^, ^12^

Transient lymphopenia is a common feature of acute viral respiratory infections.^13^ The drastic and prolonged lymphopenia of COVID-19, however, is distinctive and largely stems from falling counts of T cells.^14^, ^15^, ^16^ Regardless of the still poorly understood primary causes of this T-cell lymphopenia, the decline in T-cell count in COVID-19 demands fast and massive T-cell clonal expansion, which is TL-dependent.^17^^,^^18^ As HCTL shortens with age,^19^ T-cells from many older adults might lack the TL-dependent clonal expansion capacity required to offset the development of COVID-19 lymphopenia. These individuals, and some younger adults with inherently short HCTL, might be at a higher risk of developing COVID-19 T-cell lymphopenia and severe disease.

In absence of an acute infection, TL might exert a minimal effect on the slow T- cell turnover because the biological half-lives of circulating naïve T cells and recently formed memory T cells are ∼ 5 years and ∼ 5 months, respectively.^20^ In the face of SARS-CoV-2 infection, however, diminished TL-dependent T-cell proliferative capacity in older adults could result in a T cell shortfall.^10^, ^11^, ^12^ Moreover, the clearance of SARS-CoV-2 requires clonal expansion and differentiation of naïve T cells into SARS-CoV-2 antigen-specific effector/memory (henceforth memory) T cells.^15^^,^^16^ Short naïve T-cell telomeres might thus limit adaptive immunity against the virus even without infection-mediated T-cell lymphopenia. We therefore modelled TL-dependent T-cell clonal expansion capacity with age and virtually examined its relation to COVID-19 mortality in the general population.

## Methods

**Figure 1:**
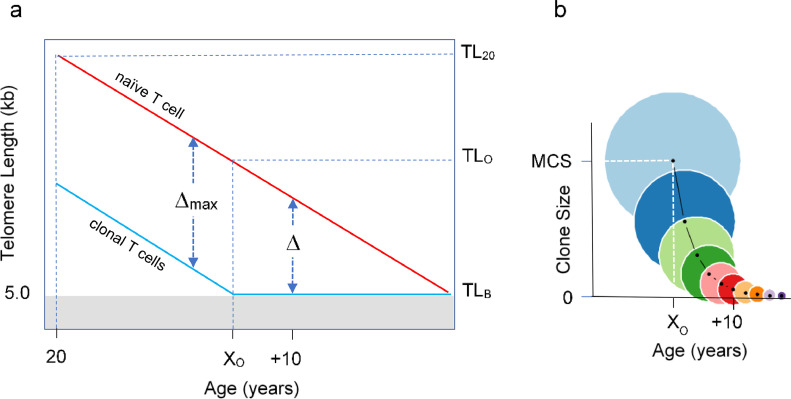
**Age-dependent T-cell telomere length (TL) and its relation to T-cell clonal expansion.** (**a**) displays age-dependent TL before () and after () clonal expansion. Naïve T-cell clonal expansion shortens telomeres by Δ, where Δ_max_ is T-cell telomere shortening resulting from expansion to form the maximal clonal size (MCS). The telomeric brink (TL_B_) of 5 kb is TL that increases the risk of cessation of replication. TL_20_ is TL at 20 years, TL_O_ is telomeric onset, which indicates the shortest T-cell TL that enables attaining MCS. X_O_ is age of onset of clonal expansion limitation. (**b**) displays T-cell clonal expansion size vs age from X_O_. Circle areas depict relative clonal size at and after X_O_. Light blue circle is MCS.

The following assumptions on T-cell replication, TL, and T-cell clone size (CS) drive our model (Figure 1): (i) T-cell TL-dependent cessation of replication is defined by a “telomeric brink” (TL_B_) that stops replication at 5 kb.^19^ (ii) TL of a naïve T cell at age 20 years (TL_20_) progressively shortens at a rate of 0.03 kb/year^21^^,^^22^ until it reaches the TL_B_. (iii) A single naïve T cell can clonally expand and through 20 successive replications it generates a maximal CS (MCS) of 2^20^ (∼ one million) memory T cells; this value was estimated based on the ∼ 1.4 kb TL difference between circulating naïve and memory T cells and the ∼ 0.07 kb telomere shortening per replication of T cells.^23^ (iv) Most memory T cells are formed in response to new antigens during childhood and early adulthood,^24^ when HCTL is comparatively long,^25^, ^26^, ^27^ enabling the achievement of MCS. (v) Due to age-dependent TL shortening, a naïve T-cell TL reaches the “telomeric onset” (TL_O_ = 6.4 kb) at “age of onset” (X_O_). Until X_O_, a naïve T cell can generate MCS. After X_O_, a naïve T cell can generate only a limited clonal size (LCS < MCS), as the TL of the clonal cells converges to the TL_B_.

Table 1 displays the parameters used to construct the model and examine its ramifications. We denote the maximal TL shortening due to clonal expansion as Δ_max_ and the TL shortening per replication as r. We define CS by the number (N) of T-cell replications producing $CS=2^{N}$. As a clone expands, telomeres of its T cells progressively shorten, i.e., Δ=rN, where r is the telomere shortening due to T-cell replication. Prior to X_O_, the maximum number of T-cell replications in clonal expansion is $N_{\max}=\Delta_{\max}/r=20$. After X_O_, the number of T-cell replications in clonal expansion is $N=\left(TL_{X}-TL_{B}\right)/r$, where X designates age and the corresponding age-dependent TL of a naïve T cell is $TL_{X}=TL_{20}-g\left(X-20\right)$, where g is the TL shortening in the naïve T cell each year. The resulting X_O_ is the number of years it takes for a naïve T cell to reach TL_O_. These onset measures are defined:

(1) $$\begin{array}{c} \begin{array}{ccc} X_{O} & = & 20+\left(TL_{20}-TL_{B}-\Delta_{\max}\right)/g \end{array}\\ \begin{array}{ccc} TL_{O} & = & TL_{B}+\Delta_{\max} \end{array} \end{array}$$

**Table 1:** Abbreviations, parameters, units, and values.

TL_20_	TL at age 20 years (kb)
TL_X_	TL at x years older than 20 years (kb)
TL_B_	Telomeric brink, stopping cell replication (5 kb)
TL_O_	TL at onset of clone size limitation (6.4 kb)
X_O_	Age in years when TL_0_ is reached
CS	Clone Size
MCS	Maximal Clone Size (∼ 10^6^ T cells)
LCS	Limited Clone Size (< MCS)
Δ_max_	TL shortening required for achieving MCS (∼ 1.4 kb)
g	TL shortening rate with age (∼ 0.03 kb/year)
r	TL shortening rate per T-cell replication (∼ 0.07 kb)
N	Number of T-cell replications in LCS (after TL_O_, X_O_)
N_max_	Number of T-cell replications in MCS (before TL_O_, X_O_)

The clone size depends on age relative to X_O_ as follows:

(2) $$\begin{array}{c} \begin{array}{ccc} X\leq X_{O} & & \mathrm{MCS}=2^{N_{\max}}=2^{\Delta_{\max}/r} \end{array}\\ \begin{array}{ccc} X>X_{O} & & \mathrm{LCS}=2^{N}=2^{\left(TL_{20}-TL_{B}-g\left(X-20\right)\right)/r} \end{array} \end{array}$$

**Figure 2:**
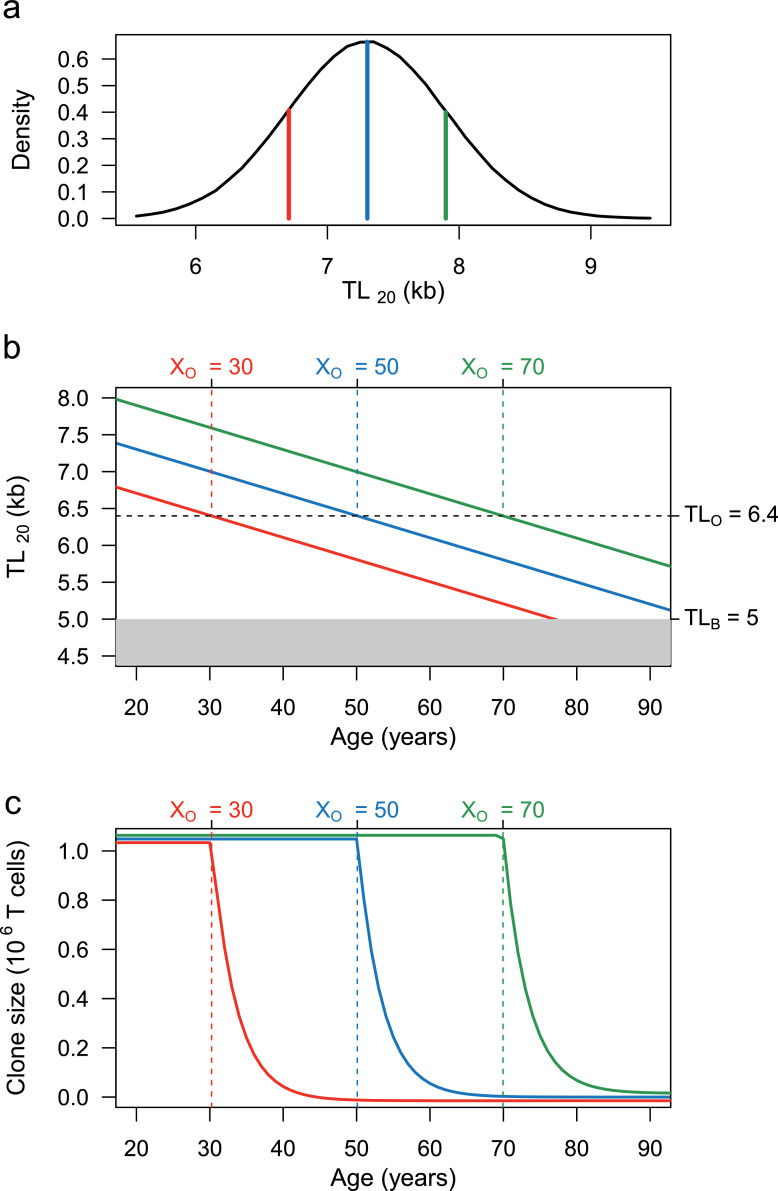
**Population distribution of T-cell TL at age 20 (TL_20_), T-cell TL shortening with age, and age-dependent change in T-cell clone size (CS).** (**a**) displays the TL_20_ distribution, showing mean TL = 7.3 kb (), long TL (mean + SD) = 7.9 kb (), and short TL (mean – SD) = 6.7 kb (). (**b**) displays age-dependent change in T-cell for mean, long and short TL_20_. Past the telomeric onset (TL_O_ = 6.4 kb), TL is insufficient to produce MCS because a full clonal expansion drops TL below the telomeric brink (TL_B_ = 5 kb). The TL_O_ is reached at different ages of onset (X_O_), i.e., an older age for T-cells with long T-cell telomeres and younger with T-cells with short telomeres. The age-dependent T-cell TL shortening (0.03 kb/year) for T cells with mean, long, and short telomeres at TL_20_ is shown by the lines. (**c**) shows that the T-cell CS is partitioned by the X_O_ into plateau and slope regions. T cells with mean, long, or short TL_20_ achieve MCS on the CS plateau, but their CS exponentially collapses (slope) once their TLs shorten below TL_O_ and exceed X_O_ (at different ages).

**Figure 3:**
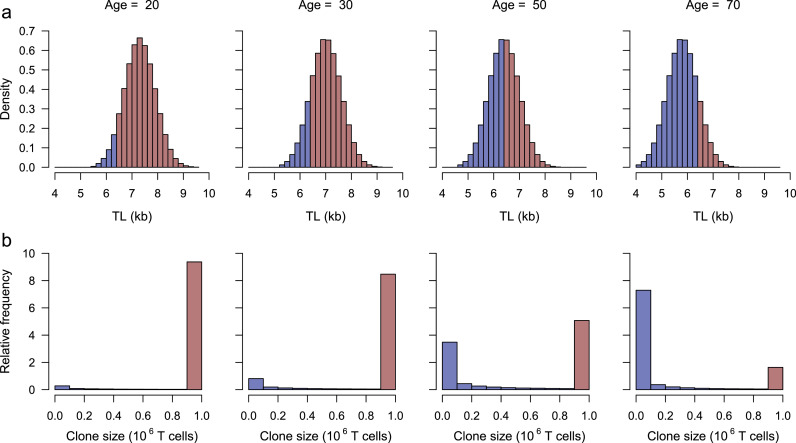
**Shifts by age in naïve T-cell TL distribution and relative frequency (0 to 10) of T-cell clone size (CS) in the population.** (**a**) displays the shift in TL_20_ distribution (Figure 2**a**) resulting from age-dependent shortening of 0.03 kb/year. It depicts TL < TL_O_ (6.4 kb) by blue bars () and TL > TL_O_ by red bars (). (**b**) displays relative frequency of CS generated by naïve T-cell clonal expansion corresponding to the categories of TL below or above TL_O_. It shows that maximal CS (MCS) of ∼ 10^6^ cells occurs in individuals with naïve T-cell TL > TL_O_, while limited CS (LCS) occurs in those with naïve T-cell TL ≤ TL_O_. At age 20, naïve T cells of nine out of ten individuals can generate MCS. At age 70, naïve T cells of less than two out of ten individuals can generate MCS, and seven out of ten generate clone sizes that are less than 0.1 MCS. At age 50 the population is approximately equally divided between the MCS and LCS groups.

Age-specific density plots of HCTL^18^ were used to extrapolate the age-dependent T-cell TL and CS with age (Figure 2), which was then used to describe the relative proportion of CS in a population (Figure 3). In Figure 3**a**, the T-cell TL distribution for age 20 was derived from 1,000,000 random generations from a normal distribution with a mean = 7.3 kb and SD = 0.6 kb.

**Figure 4:**
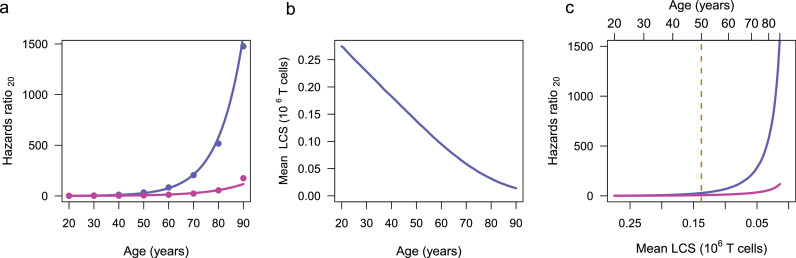
**Steps linking mean limited clone size (LCS) to COVID-19 mortality and general mortality hazards ratios_20_ in the population.** (**a**) displays data based on COVID-19 mortality () and non-COVID-19 mortality (), and corresponding exponential fitted relationships for hazards ratios_20_ ( and ). (**b**) displays the relationship of mean LCS in units of 10^6^ cells with age, generated with Eq. 2, using the TL_20_ distribution of Figure 2**a**. (**c**) displays the relationships of hazards ratios_20_ generated from COVID-19 mortality and non-COVID-19 mortality plotted against mean LCS obtained from Figure 4**b**. The top of the panel also displays age. The divergence between the COVID-19 and non-COVID-19 mortalities occurs at mean LCS of ∼ 0.13×10^6^ T cells. At the corresponding age, 50 years, the population is about evenly divided into the LCS and MCS groups (Figure 3**b**). After this age, increased proportion of the population is in the LCS group, which is susceptible to COVID-19 mortality, whereas the MCS group is not.

The link of the mean LCS to COVID-19 and general mortality in the population (Figure 4) was developed by calculating age-specific COVID-19-linked mortalities and total non-COVID-19 linked mortalities normalized by the age-specific US population. The hazards ratio, defined hazards ratio_20_ = (mortality_age_/population_age_) / (mortality_20_/population_20_), is based on the CDC records of 494,234 provisional COVID-19 deaths and 3,845,819 total deaths between January 1, 2020, through March 8, 2021^28^ and the 2019 US Census.^29^ Non-COVID-19 mortalities were estimated by subtracting the COVID-19 mortalities from the total mortalities for each age group. The comparison of the COVID-19 and non-COVID-19 hazards ratios_20_ (Figure 4**a**) assumes that TL-dependent COVID-19 mortality only occurred for CS < MCS. Effect of other LCS cut-off levels, e.g., < 0.5 MCS or < 0.15 MCS on the relationship of LCS, hazards ratio_20_ and age is explored in *Supplementary Information A1* and the uncertainty in the onset of clonal expansion limitation is explored in *A2*.

The following considerations are relevant for appraising the model's parameters and assumptions: First, while the MCS is based on the *in vivo* TL difference between naïve and memory T cells, the data on telomere shortening per T-cell replication are from cultured cells.^23^ A similar approach (based on data from circulating hematopoietic cells and telomere shortening in cultured cells) was previously used to generate consistent information on hematopoietic cell replicative kinetics.^30^^,^^31^ Second, the model's TL population distribution is derived from a large population-based study^19^ that measured HCTL by Southern blotting.^32^ Its telomere data are consistent with another large-scale study that used Flow-FISH to measure HCTL.^25^ Third, the model is based on age-dependent shortening of HCTL and not T-cell TL. As TL differences among leukocyte lineages within the individual are far smaller than the inter-individual HCTL variation,^33^ T-cell TL largely mirrors HCTL. Fourth, the TL signal for cessation of cell replication originates from the shortest telomeres in the nucleus and not their mean TL.^34^^,^^35^ Using the Telomeres Shortest Length Assay (TeSLA), a method that tallies and measures the shortest telomeres,^36^ a recent study showed that in patients with COVID-19 the shortest telomeres in peripheral blood mononuclear cells were associated with low lymphocyte counts.^12^ The principles that drive our model thus likely apply to the T cell's shortest telomeres.

### Ethics statement

The paper used data from publicly available databases and involved no animal or human experiments or studies.

### Statistics statement

Analysis of model sensitivity and parameter uncertainty is provided in **Supplementary Information A1**.

### Role of the funding source

The study sponsors had no role in study design, collection, analysis, and interpretation of the data. Funding sources had no contribution to writing the report or decisions on publication.

## Results

The core conclusion of the model is as follows: Until reaching the age of onset, X_O_, age-dependent shortening of T-cell telomeres exert little influence on the ability of naïve T cells to generate the MCS of 2^20^ memory T cells in response to antigen stimulation (Figure 1**a**). However, once X_O_ is reached, the ability of T cells to expand clonally declines in an exponential manner, i.e., 2^20^ → 2^19^→ 2^18^ → 2^17^, etc. Since T-cell TL shortens at a pace of 0.03 kb/year and by 0.07 kb/replication, the T-cell clonal expansion capacity drops by half every 2.3 years past X_O_. Thus, in one decade after X_O_, the clonal expansion capacity of naive T cells is about 5% of the MCS (Figure 1**b**).

HCTL tracks with age, meaning that as they get older, individuals maintain their TL ranking at any given age.^37^ To examine the effect of inter-individual TL variation and age on T-cell clonal expansion, consider three individuals with average, long (one SD above the mean) and short (one SD below the mean) naïve T-cell TL_20_ (Figure 2**a**): The individual with average TL_20_ can attain the MCS of ∼10^6^ cells up to age 50 years, i.e., the individual's X_O_ (Figure 2**b**). Thereafter, while the individual's naïve T cells continue their slow age-related telomere shortening (Figure 2**b**), their clonal expansion capacity declines exponentially from the MCS (Figure 2**c**). Next consider the individual with long T-cell TL_20_ (Figure 2**a**). The ability of naive T-cells of this individual to achieve MCS extends X_O_ to 70 years (Figure 2**c**). In contrast, naïve T cells of the individual with short T-cell TL_20_ are only able to achieve MCS to the X_O_ of 30 years (Figure 2**c**). Our model thus showcases the high X_O_ variability across the population.

The proportion of the population with T-cell TL > TL_O_ slowly and continuously declines from 90% at age 20 to 10% at age 70 (Figure 3**a**). In contrast, after reaching X_O,_ the T-cell clonal expansion capacity rapidly declines with age, and therefore the general population divides into two groups when the mean population X_O_ is 50 years: a MCS group that can generate a full clonal expansion of naïve T cells and a LCS group that can generate only a fraction of the MCS (Figure 3**b**).

What then might be the minimal TL-dependent T-cell CS that enables survival of an individual contracting COVID-19? The definitive answer awaits telomere and T-cell data in populations of COVID-19 patients. That said, we infer this CS from a comparison of age-dependent hazards ratio of mortality relative to age 20 years (hazards ratio_20_) from COVID-19 and from general causes other than COVID-19. The hazards ratio_20_ increased exponentially with age for both COVID-19 mortality and non-COVID-19 mortality, but mortality from COVID-19 increased much faster than non-COVID-19 mortality (Figure 4**a**).

We assumed that, as a group, individuals who generate MCS experience no T-cell TL-related COVID-19 mortality and accordingly focused on the mean CS for the LCS group, i.e., individuals older than X_O_ (Figure 3**a**). The TL-limited clonal expansion of these individuals, we assumed, might contribute to their propensity to die from COVID-19, given the association between T-cell lymphopenia and COVID-19 mortality. Figure 4**b** shows that the mean LCS in individuals older than X_O_ decreases in a near linear manner with age. Plots of the mean LCS vs. hazards ratio_20_ of COVID-19 mortality and non-COVID-19 mortality suggest a divergence between the two trajectories during the 6th decade (Figure 4**c**). The figure also displays the mean LCS at the age of 50 years, when the size of LCS group is equivalent to that of the MCS group (Figure 3**b**). Mean LCS at this age amounts to ∼ 0.13 × 10^−6^ T cells.

## Discussion

Apart from heritability,^38^ no other single factor so profoundly affects HCTL as does ageing, explaining the key conclusion of our model: Age-dependent telomere shortening might drain T cells of much needed clonal expansion capacity in the face of SARS-CoV-2 infection. As SARS-CoV-2 memory T cells play a greater role than neutralizing antibodies in recovering from the infection,^15^ the ageing effect on HCTL could impede adaptive immunity and heighten the risk for severe COVID-19. Moreover, we assume that the MCS applies not only for naïve T cells that clonally expand to produce memory T cells but also formation of new naïve T cells. This means that regardless of the primary cause of COVID-19 T-cell lymphopenia, the T cell response after X_O_ will be compromised on two levels, i.e., formation of SARS-CoV-2-specific memory T-cells and replenishing the loss of naïve T-cells.

Mortality (yes/no) is a clear outcome of COVID-19. In contrast, ‘severe’ COVID-19 is an amorphous outcome that is categorized differently in various studies. Moreover, data of severity of COVID-19 are not uniformly accessible from public records as are mortality data. Therefore, we have elected to use mortality as an ‘endpoint’ in our model. The divergence of COVID-19 mortality from non-COVID-19 mortality when the mean LCS is about one tenth of MCS suggests the following: In the absence of COVID-19, ∼ 10% MCS is generally sufficient to accommodate the low turnover of T-cells.^20^ This LCS, however, might contribute to mortality in the face of SARS-CoV-2, because the infection demands massive T-cell clonal expansion to offset the primary cause of the dropping naïve T-cell count, and to generate memory T-cells that clear the virus.

As illustrated in Figs. 2 and 3, our model might also apply to naïve T cells of a subset of younger adults, whose HCTL is ranked at the lower range of the HCTL distribution in the general population.^19^^,^^25^ Comparatively short HCTL might also diminish naïve T-cell clonal expansion in response to SARS-CoV-2 infection in males, whose HCTL is shorter than in females from birth onwards,^19^^,^^25^^,^^27^^,^^38^^,^^39^ persons with atherosclerotic cardiovascular disease,^39^, ^40^, ^41^ obese persons^42^^,^^43^ and smokers^43^, ^44^, ^45^ whose HCTL is respectively shorter than that in healthy, lean and non-smoking individuals. These groups of individuals are at a higher risk of severe COVID-19 and death from the disease.^46^, ^47^, ^48^, ^49^ There are, of course, factors other than HCTL that contribute to the propensity of these groups to severe COVID-19. The X_O_/TL_O_ concept provides, however, the framework for testing the role of HCTL in the pathogenesis of COVID-19 regardless of age and comorbidities.

Humans have comparatively short telomeres relative to their long lifespan,^50^^,^^51^ and therefore our model may not apply to most terrestrial mammals, including laboratory animal models that are used for viral research. For instance, TL-mediated replicative ageing is probably inconsequential during the 2, 3-year lifespan of mice, considering their long telomeres (mean TL > 30 kb) and robust activity in their somatic cells of telomerase, the reverse transcriptase that elongates telomeres. In contrast, the average human TL at birth is only ∼ 9.5 kb.^27^ As telomerase activity is repressed in replicating human somatic cells, their short telomeres experience further age-dependent shortening after birth. Although naïve T cells have some telomerase activity, it is insufficient to prevent their age-dependent telomere shortening, and ageing may thus undermine the T-cell clonal expansion in many older humans.

Relatedly, the model shows that individuals with naïve T-cells whose TL_20_ is one SD below the mean might be unable to achieve MCS as early as the third decade of life. This unexpected finding suggests that these individuals might (a) develop severe COVID-19 T-cell lymphopenia despite their young age, or (b) tap more naïve T cells for clonal expansion in response to exposure to pathogens.^52^ Older adults may not have sufficient naïve T cells, particularly, naïve CD8 T cells, for this purpose.^53^ Empirical data based on HCTL measurements in otherwise healthy adults who developed COVID-19 will help testing these alternatives.

We acknowledge limitations: The model draws on HCTL data from populations comprising principally whites of European ancestry in high-income countries. It should also be tested in populations of different ancestries and in low- and middle-income countries. That said, we anticipate that the principles of the model will hold, although the X_O_ and TL_O_ might shift up or down depending on specific populations and geographical regions. The TL difference between naïve T cells and memory T cells likely reflects the clonal expansion in response to not only a single encounter but multiple encounters with a given antigen and its cross-reactive antigens. Thus, the MCS and LCS definitions in absolute T-cell numbers might be off the mark. Of note, however, the MCS and LCS can be expressed in the model in relative units of MCS (i.e., 0.5 MCS, 0.25 MCS, etc.) rather than absolute units (numbers of T cells), yielding identical results. Therefore, the principles of our model are likely to hold notwithstanding the above limitations. Finally, our model focuses on telomere length dynamics in limiting T-cell clonal expansion through mechanisms that trigger replicative senescence. However, independent mechanisms might limit T-cell clonal expansion through pathways that lead to T-cell exhaustion. Typically, this exhaustion is associated with the upregulation of the programmed cell death (PD-1) pathway,^54^ but de novo DNA-methylation might also promote T cell exhaustion.^55^

In conclusion, while our model is clearly an oversimplification, it highlights an overlooked effect of ageing within the very complex system of T-cell regulation.^53^ The insight generated by our model might set the stage for measurement of TL parameters not only in older adults but also the general population, helping to identify, regardless of age, individuals vulnerable to severe COVID-19 because of short T-cell telomeres. These individuals might show, in addition, an early waning immunity after SARS-CoV-2 vaccination, facilitating the evolution of novel variants of the virus.^56^ Finally, the ramifications of these conclusions go beyond the influence of TL on T-cell response to SARS Cov-2 infection and vaccination against the virus. They suggest that TL might be a limiting factor in immunotherapies whose efficacies depend on clonally expanding (*in vivo* and *in vitro*) transplanted hematopoietic cells, chimeric antigen receptor T cells, and tumour-infiltrating lymphocytes.

## Declaration of interests

The authors declare no competing interests.

## Appendix Supplementary materials

Image, application 1

*Supplementary Information:* Supplementary Information A contains Text, figures, and table. Supplementary Information B contain R programming language scripts for generating the model and Figs. Figure 2, Figure 3, Figure 4, S1, and S2. Image, application 2
