# Supplementary material for: Telomere-length dependent T-cell clonal expansion: A model linking ageing to COVID-19 T-cell lymphopenia and mortality
Source: eBioMedicine. 2022 Apr 1;78:103978. doi: 10.1016/j.ebiom.2022.103978 (PMC8970968; doi:10.1016/j.ebiom.2022.103978)
Supplement: Supplementary file 1 [file mmc1.pdf]

Supplementary Information File A for

Telomere-length dependent T-Cell clonal expansion: a model linking ageing to COVID-19 T-Cell lymphopenia and mortality

James J. Anderson*, Abraham Aviv, Ezra Susser, Konstantin G. Arbeev, Anatoliy I. Yashin, Daniel Levy[6], Simon Verhulst

*Corresponding author. Email: jjand@uw.edu

This file includes:

Supplementary Text A1 and A2

Figs. S1 to S2

Tables S1

References (1-4)

Other Supplementary Information for this manuscript includes the following:

Supplementary_Information_B.R   R code (1) to make model and Figures 2, 3, 4, S1, S1

Text A1. Effect of limited clone size (LCS) definition on model response and correlation with observed mortality

The model assumes that mortality from COVID-19 only occurs when T-cell clonal expansion cannot achieve maximum clone size (MCS). We were unable to *a priori* select how LCS might affect mortality. We explored instead the relationship of mean LCS with years and the Hazards ratio_{20} for three cutoff levels used for defining LCS: (I) LCS < MCS, (II) LCS < 0.5 MCS, and (III) LCS < 0.15 MCS (**Figure S1**). In cutoff I, any clone that cannot achieve a MCS is included in the LCS subpopulation and therefore susceptible to SARS-CoV-2 mortality. In cutoff II, only clones that are less than half the MCS are included in the LCS subpopulation and in cutoff III only clones that are less than 15% of the MCS are included.

The percent of the subpopulation in the LCS category (%LCS) increases from ~ 5% at age 20 to ~ 90% at age 90 (**Figure S1a**). These percentages change little between the three cutoff levels. The maximum difference in % LCS in the definitions occurs at age 50 years. This close tracking with age indicates that the LCS cutoff has a minimal effect on the partition of the subpopulations in **Figure 3a,b**. Note that the height of the histogram bars in **Figure 3b** does not depend on the LCS cutoff.

Figure S1b illustrates that the LCS cutoff has a strong effect on the mean LCS pattern with age. But for all cutoffs, the mean LCS declines in a nearly linear manner with age. Thus, the assumption of a linear age-dependence of mean LCS with age is unaffected by the LCS cutoff choice.

Figure S1c depicts the effect of the LCS cutoff on the hazard ratio_{20}. Here, because the cutoffs do not significantly affect linearity of mean LCS with age, they rescale the mean LCS at which the hazards ratio_{20} of COVID-19 and non-COVID-19 diverge, which for **Figure 4c** is taken as the critical hazards ratio_{20} = 25.4. Then the mean LCS associated with this critical hazards ratio_{20} linearly scales with the LCS cutoff as $\overline{\text{LCS}}_{\text{crit}} = 0.142\text{LCS}_{\text{cutott}}$.

In sum, the cutoff level changes the critical mean LCS at which the COVID-19 and non-COVID-19 hazards ratios_{20} diverge but this critical level is proportional to the cutoff level. Thus, a significant divergence of COVID-19 and non-COVID-19 hazards ratios_{20} is robust to the definition of the CS level susceptible to mortality.

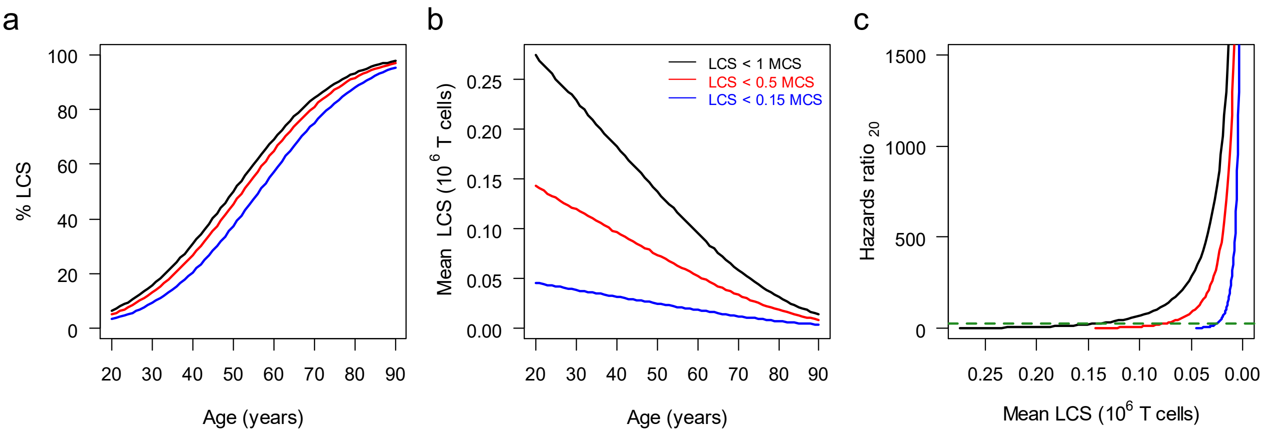

Figure S1. Model properties with three LCS cutoff levels: LCL < 1.0 MCS (—), LCL < 0.5 MCS (—) and LCL < 0.15 MCS (—). (a) % of population in LCS group by age for cutoff levels, (b) mean LCS vs age for cutoff levels, (c) hazards ratio$_{20}$ vs mean LCS for cutoff levels. Dashed line (– –) depicts hazards ratio$_{20}$ (25.4) at which COVID-19 and non-COVID-19 mortalities diverge.

Text A2. Effects of parameter estimates on model response

Parameters (MCS, LCS, X_O, TL_O, g, r, TL_B, TL_{20}, and Δ_{max}, **Table 1** in the paper) were derived from published data (**Table S1** shows references). To demonstrate the contribution of these terms to the model uncertainty, we use the propagation of error approach assuming uncorrelated terms. Based on the equation $X_O = 20 + \left(TL_{20} - TL_B - \Delta_{max}\right)/g$ the uncertainty in the TL_O measures is

$$\begin{aligned} \sigma^2_{X_O} &\approx \frac{1}{g^2}\left(\sigma^2_{TL_B} + \sigma^2_{\Delta_{max}} + \left(X_O - 20\right)^2 \sigma^2_g\right) \\ \sigma^2_{TL_O} &= \sigma^2_{TL_B} + \sigma^2_{\Delta_{max}} \end{aligned} \quad \text{(S1)}$$

To estimate uncertainty of CS, first define the number of T-cell replications in clone expansion as $N_X = \left(TL_{20} - g\left(X - 20\right) - TL_B\right)/r$ where age is X. Then variance in replications is

$$\sigma^2_{N_X} \approx \frac{1}{r^2}\left(\sigma^2_{TL_B} + \left(X - 20\right)^2 \sigma^2_g + N_X^2 \sigma^2_r\right) \quad \text{(S2)}$$

Next, to estimate uncertainty in CS, define the clone size in terms of the number of replications as $CS = 2^{N_X}$, then the variance in the CS for a clone with limited expansion capacity is

$$\sigma^2_{LCS_X} \approx \left(\ln(2)2^{N_X}\right)^2 \sigma^2_{N_X} \qquad \text{(S3)}$$

and the variance on MCS is calculated from Eq. (S3) by setting X to X_O giving

$$\sigma^2_{MCS} \approx \left(\ln(2)2^{N_{X_O}}\right)^2 \sigma^2_{N_{X_O}} \qquad \text{(S4)}$$

The uncertainties for these measures are calculated using the parameter estimates and standard deviations given in **Table S1**.

The uncertainty in the TL of the TL_O from Eq. (S1) is σ_{TL_O} = 0.1 kb, or 1.5%. The uncertainty in X_O depends on TL_{20}. **Figure S2a** shows that the uncertainty varies slightly with TL_{20}. Using the population mean TL_{20} of 7.3 kb the uncertainty is $\sigma_{X_O} \sim 3.4$ years.

The uncertainty in CS and number of replications depend on age X (**Figure S2b,c**). In the figure, the uncertainty is normalized by measures prior to TL_O, i.e., $N_{max} = 20$ and MCS = 2^{20}. In panels **b** and **c**, the relative uncertainties decrease after the TL_O, here set to X_O = 50 years to represent individuals with average TL_{20} of 7.3 kb. Notably, the large uncertainty in the MCS is wholly dominated by σ_r through Eq. (S2). While the uncertainty in CS is significant, the properties of the model are expressed in terms of the ratio CS/MCS and not the actual value of MCS.

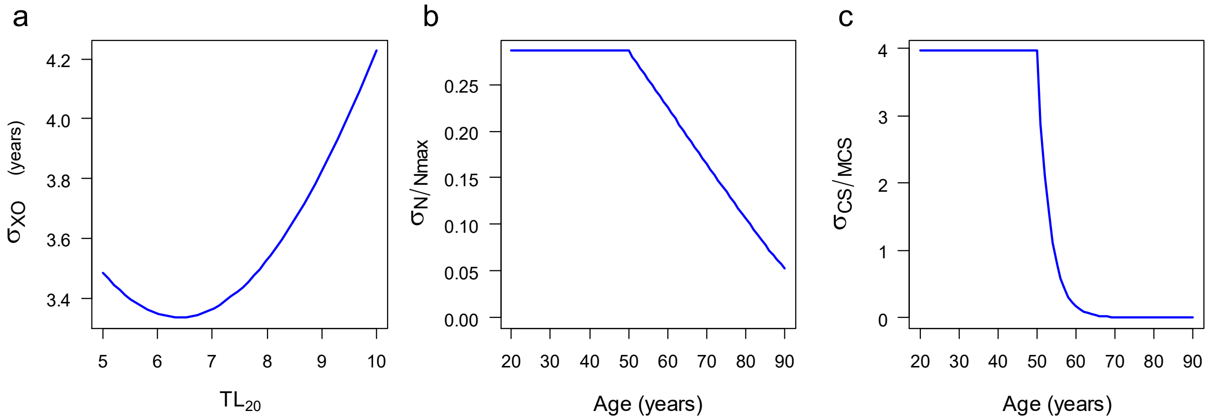

Figure S2. Uncertainty in parameter estimates. (a) Uncertainty in X_O with TL_{20}. (b) Uncertainty in clone replication number (N) with age normalized by N_{max}. (c) Uncertainty with age in CS normalized by the MCS.

Table S1. Parameter estimates and uncertainties.

parameter	value	units	Reference
$\sigma_{\Delta_{max}}$	0.1	kb	2
σ_B	0.0034	kb	3, 4
σ_g	0.00065	kb/year	2
σ_r	0.02	kb/replication	4
Δ_{max}	1.4	kb	4
TL_B	5	kb	2
TL_{20}	5-9	kb	5
g	0.03	kb/year	2
r	0.07	kb/replication	2

SI References

1. R Core Team. R: A language and environment for statistical computing. Vienna, Austria: R Foundation for Statistical Computing; 2021.

2. Steenstra T, Hjelmborg JV, Kark JD, Christensen K, Aviv A. The telomere lengthening conundrum--artifact or biology? Nucleic Acids Res. 2013;41(13):e131.

3. Aviv A, Hunt SC, Lin J, Cao X, Kimura M, Blackburn E. Impartial comparative analysis of measurement of leukocyte telomere length/DNA content by Southern blots and qPCR. Nucleic Acids Res. 2011;39(20):e134-e.

4. Weng NP, Hathcock KS, Hodes RJ. Regulation of telomere length and telomerase in T and B cells: a mechanism for maintaining replicative potential. Immunity. 1998;9(2):151-7.

5. Steenstrup T, Kark JD, Verhulst S, Thinggaard M, Hjelmborg JVB, Dalgård C, et al. Telomeres and the natural lifespan limit in humans. Aging. 2017;9(4):1130-42.
